# Intranasal and Oral Pharmacological Sedation in Pediatric Dentistry: A Thematic Scoping Review of Efficacy, Recovery, and Safety Outcomes

**DOI:** 10.3390/children13091153

**Published:** 2026-08-27

**Authors:** Ayman M. Sulimany, Hebah M. Hamdan, Mannaa K. Aldowsari, Saad Bin Saleh

**Affiliations:** 1 Department of Pediatric Dentistry and Orthodontics, College of Dentistry, King Saud University, Riyadh 11545, Saudi Arabia; maldowsari@ksu.edu.sa (M.K.A.); ssbinsaleh@ksu.edu.sa (S.B.S.); 2 Bio-Research Chair in Dental Health, College of Dentistry, King Saud University, Riyadh 11545, Saudi Arabia; 3 Department of Periodontics and Community Dentistry, College of Dentistry, King Saud University, Riyadh 11545, Saudi Arabia; hhamdan1@ksu.edu.sa

**Keywords:** conscious sedation, intranasal sedation, oral route, thematic analysis, combination drugs, special healthcare needs children

## Abstract

**Highlights:**

**What are the main findings?**

- In most included studies, intranasal sedation showed shorter recovery than oral routes; onset varied markedly across agents and studies, ranging from approximately 9 to 41 min.
- Thematic analysis developed six major themes covering drug efficacy, safety, onset, recovery, patient acceptance, and special need populations.

**What is the implication of the main findings?**

- The non-invasive nature of intranasal delivery, with shorter recovery reported in most studies, makes it a practical option to improve cooperation in anxious or uncooperative children, although onset and sedation success vary by agent, and evidence for added benefit from multi-drug regimens remains mixed.
- Because recovery time, patient acceptance, and adverse event profiles vary widely across agents and routes, sedation should be individualized to the child’s age, behavior, and special healthcare with appropriate monitoring, underscoring the need for standardized, evidence-based protocols to guide safe drug and route selection.

**Abstract:**

Introduction: Conscious sedation (CS) is commonly recommended in pediatric dental clinics to manage uncooperative and anxious patients. Various routes of drug administration have been introduced to achieve effective CS. Among these, the intranasal (IN) route has gained popularity in dentistry due to its non-invasive nature. However, there is currently no comprehensive thematic review analyzing the efficacy and safety of drug regimens delivered via the intranasal route alone or in combination with other administration routes. Therefore, the aim of the present review is to thematically analyze the efficacy and safety of different drug regimens used for conscious sedation delivered through the intranasal route and/or in combination with other routes. Material and Method: This scoping review was conducted and reported in accordance with the Preferred Reporting Items for Systematic Reviews and Meta-Analyses extension for Scoping Reviews (PRISMA-ScR) checklist. The protocol was prospectively registered on the Open Science Framework (OSF) prior to data extraction. Predefined inclusion criteria were developed, and five electronic databases were searched. The search identified a total of 239 articles from different databases. After evaluation and removal of duplicates, a total of 54 articles were assessed for full-text eligibility. Finally, 18 articles were included, and data extraction was performed. To develop themes the included articles were imported to the N-vivo software package. Results: Six themes and sixteen subthemes were developed. The major themes were route efficacy and behavioral outcomes, onset of action, patients’ acceptance and tolerance, special needs population, recovery and duration, safety profile and adverse events. Conclusion: Combined intranasal and oral conscious sedation is a generally safe and well-accepted approach in pediatric dentistry, but variations in recovery time, patient acceptance, and adverse events necessitate individualized sedation strategies.

## 1. Introduction

The American Society of Anesthesiologists defines conscious sedation (CS) as an anesthetic procedure that helps uncooperative patients relax and respond to verbal commands, either alone or with the help of light stimulation [1]. The main goal of CS is to minimize physical discomfort and pain and ensure the safe completion of any surgical or non-surgical procedures [2].

Different pharmacological agents such as midazolam, ketamine, dexmedetomidine, and nitrous oxide are administered either alone or in conjunction with other drugs to achieve CS [3]. The most commonly used CS agent in dentistry is midazolam. Studies have reported different techniques to deliver CS amongst children, including intranasal sedation, inhalation sedation, oral sedation, and intravenous sedation [4,5,6]. Despite different techniques and their proven efficacy, they have all been proven safe in dentistry [4]. In 2018, intranasal sedation (INS) gained popularity in dental clinics because of its easy administration, fewer side effects, and faster delivery [4,7]. Moreover, studies have reported that INS is the most reliable alternative to other routes of CS administration [3]. This does not reduce the established role of inhalation sedation with nitrous oxide, which remains the safest and most predictable conscious sedation technique in dental practice and is generally the first-line choice where it is available and effective; intranasal sedation is best regarded as complementary, with particular utility where inhalation sedation is refused, poorly tolerated, or insufficient.

Recent reviews have described a shift towards multi-drug sedation regimens intended to optimize behavioral outcomes, while emphasizing that safety profiles vary with the agents selected, patient characteristics, and clinical protocol [8,9,10]. Combined approaches, including intranasal dexmedetomidine with oral midazolam, have attracted particular attention in this context [10].

Studies have reported that regular dental treatment under LA is challenging for children with special needs [11]. Hence, to maintain their behavior, there is a need for CS. INS is considered to be the better option for children with special needs, as there is no requirement for any oral or IV regimen, which might increase the anxiety of children [12]. Moreover, managing children with special healthcare needs is challenging for the entire dental team. Many organizations have published guidelines regarding the use of appropriate sedation and general anesthesia for both healthy and special needs children [1,13]. Despite challenges encountered by the dental staff in managing these children, there is no comprehensive analysis explaining the requirements and guidelines for using INS on children with special healthcare needs. Moreover, there is considerable heterogeneity in reported drug combinations, dosage regimens, outcome measures, and safety monitoring across studies. Moreover, qualitative insights such as clinician preferences, perceived barriers, parental satisfaction, and practical challenges in administration are often underrepresented in quantitative syntheses. This gap complicates clinical decision-making and hinders the development of standardized, evidence-based guidelines.

To address these gaps, this scoping review aims to systematically map the existing evidence on intranasal, oral, and other routes of sedation and their combinations used in pediatric dentistry. Unlike previous reviews that focused primarily on efficacy or adverse events, this review will adopt a dual approach by (1) cataloging the range of pharmacological regimens and their reported outcomes and (2) conducting a thematic analysis.

By synthesizing both quantitative and qualitative evidence, this review seeks to provide a comprehensive landscape of current practices, identify consistent patterns and recurring concerns, and illuminate key knowledge gaps. The findings are intended to inform clinical practice, guide future research, and serve as a foundation for subsequent systematic reviews or clinical trials aimed at optimizing sedation protocols for pediatric and special needs dental patients.

## 2. Materials and Methods

This scoping review protocol was developed in accordance with the Joanna Briggs Institute (JBI) methodological framework for scoping reviews [14]. This review was conducted and reported in full compliance with the Preferred Reporting Items for Systematic Reviews and Meta-Analyses extension for Scoping Reviews (PRISMA-ScR) checklist (Supplementary Table S1). The protocol was prospectively registered on the Open Science Framework (OSF) prior to data extraction (Registration: https://osf.io/3f5d6, accessed on 19 June 2026), ensuring transparency, replicability, and minimizing reporting bias.

### 2.1. Review Aim and Questions

The primary aim of this scoping review is to systematically map the existing literature on intranasal, oral, and other routes of sedation and their combinations used for procedural sedation in pediatric dentistry, and to conduct a thematic analysis of the qualitative and experiential data embedded within this literature.

Guided by the JBI Population, Concept, Context (PCC) framework, the review seeks to address the following questions:

What pharmacological agents, administered via IN and/or PO routes combinations, have been investigated for procedural sedation in pediatric and special needs dental patients?

#### 2.1.1. PCC Framework

Population (P): Children and adolescents and/or special needs children, aged 0 to 18 years, undergoing diagnostic or therapeutic dental procedures. Concept (C): The administration of one or more pharmacological agents, as monotherapy or in combination (intranasal and/or oral), for the purpose of achieving conscious or procedural sedation, where at least one agent is delivered via the intranasal and/or the oral route. Context (C): Clinical dental settings globally, including primary care clinics, hospital dental departments, and academic institutions.

#### 2.1.2. Eligibility Criteria

Eligible studies enrolled pediatric populations (0–18 years, or participants explicitly described as children or adolescents), including children with special healthcare needs, undergoing dental procedures. Eligible interventions comprised any pharmacological sedation regimen in which at least one sedative agent was administered by the intranasal (IN) route and/or the oral route, the latter encompassing oral enteral (PO, swallowed) and oral transmucosal (buccal, sublingual) administration. Regimens combining agents by the same route and regimens combining IN and oral routes were both eligible. Studies comparing doses of a single agent were eligible and classified as monotherapy. All primary research designs were considered, including randomized controlled trials, non-randomized controlled trials, prospective and retrospective cohort studies, case–control studies, cross-sectional studies, and qualitative or mixed-methods studies. Studies were required to report at least one relevant outcome, such as physiological parameters (vital signs or oxygen saturation), behavioral or sedation-related outcomes (sedation scores or cooperation), time-based measures (onset, duration, or recovery), adverse events, or qualitative accounts of patient, parent, or provider experiences. Only peer-reviewed articles published between January 2010 and November 2025 were included. Articles published in English were eligible, while studies in other languages were screened at the title and abstract level and included for full-text review if appropriate translation resources were available.

#### 2.1.3. Exclusion Criteria

Studies focusing on non-dental medical or surgical procedures were excluded, as were those conducted exclusively in adult populations (>18 years of age). Pre-clinical investigations, including in vitro studies and animal models, were not eligible for inclusion. Additionally, pharmacokinetic studies that did not report clinical outcomes, as well as secondary literature such as narrative reviews, editorials, commentaries, and opinion pieces, were excluded.

#### 2.1.4. Information Sources and Search Strategy

A comprehensive multi-database search strategy was developed in collaboration with a senior health sciences librarian to ensure optimal sensitivity and specificity. The primary electronic databases searched included PubMed, Embase, the Cochrane Central Register of Controlled Trials (CENTRAL), Scopus, and the Web of Science Core Collection. In addition to database searching, forward and backward citation tracking (snowballing) was conducted for all included studies and for key systematic reviews identified during the search process. The search strategy incorporates a combination of controlled vocabulary terms (Medical Subject Headings [MeSH] and Emtree) and free-text keywords corresponding to the core Population, Concept, and Context (PCC) elements (Table 1). The strategy was initially developed for Ovid MEDLINE and subsequently adapted for use in the other databases by modifying syntax and subject headings as appropriate. Boolean operators (AND, OR) and adjacency operators were employed to combine search terms effectively. The complete search strategy, including all MeSH terms, free-text keywords, Boolean operators, and date filters applied, is provided in full in Supplementary Table S2. All searches were executed on 31 January 2026; the date limit applied was 1 January 2010 to 30 November 2025.

#### 2.1.5. Selection of Sources of Evidence

All identified records were imported into Excel and EndNote 20 for deduplication and screening. The selection process was conducted in two sequential stages, independently by two reviewers (A.M.S and S.B.S). In the first stage, titles and abstracts were screened against the eligibility criteria to identify potentially relevant studies. In the second stage, the full texts of these articles were retrieved and assessed for final inclusion. Any discrepancies at either stage were resolved through discussion between the two reviewers, with a third senior reviewer consulted if consensus could not be reached.

#### 2.1.6. Data Charting Process

Data was extracted independently by two reviewers (A.M.S and S.B.S) using a standardized, pilot-tested data charting form in Microsoft Excel. Extracted data included domains like study identification details such as author(s), year of publication, and country; methodological characteristics, including study design, setting, sample size, and participant demographics; and intervention details, encompassing specific drug names, dosages (mg/kg), routes of administration (IN or PO), sequence and timing of administration, and the use of any adjunctive agents or behavioral techniques. Information on comparators, including details of any control or comparison interventions, was also recorded. Furthermore, all reported outcomes and their measurement methods were extracted, including specific definitions and scales used. Finally, key quantitative results relating to efficacy, safety, timing, and adverse event rates were documented.

#### 2.1.7. Thematic Analysis of Qualitative Evidence

Textual data extracted from the “Qualitative and Interpretive Data” domain were analyzed using a reflexive thematic analysis approach, following the methodology described by Braun and Clarke [15], with the process facilitated using the NVivo software package (Nvivo v.12.2 (nvivo.software.com/12.2/)). Reviewers first familiarized themselves with the extracted text to gain an in-depth understanding of the data. Two reviewers (A.M.S and S.B.S) then independently generated initial codes in an inductive, data-driven manner. These codes were collated and examined to identify potential descriptive themes. The themes were subsequently reviewed, refined, named, and clearly defined to ensure they accurately represented the dataset. Finally, the thematic framework was integrated and reported both narratively and visually.

## 3. Results

**Figure 1:**
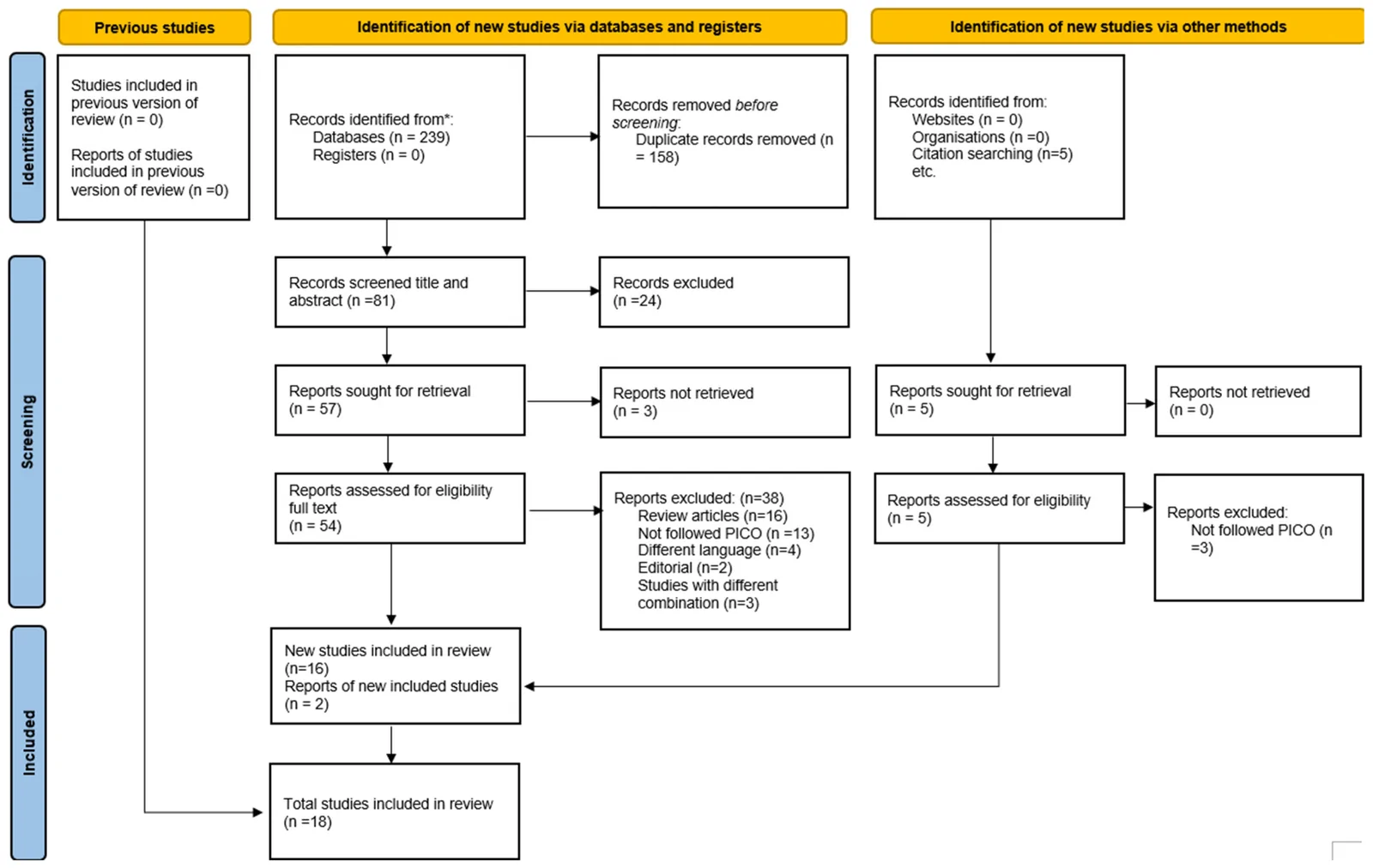
PRISMA flowchart [14]. * Electronic databases like PubMed/Medline, Scopus, Web of Science, Embase.

A systematic multi-database search of PubMed, Embase, the Cochrane Central Register of Controlled Trials (CENTRAL), Scopus, and the Web of Science Core Collection resulted in total of 239 articles. Following automated and manual deduplication using EndNote 20, 158 duplicate records were removed. Eight-one records remained and were screened against the title and abstract, of which 24 were excluded for not meeting the eligibility criteria. Fifty-seven reports were sought for retrieval; four could not initially be obtained, of which one was subsequently provided by the corresponding author, leaving three reports not retrieved and 54 reports assessed for full-text eligibility. After applying the predefined eligibility criteria, 38 studies were excluded for the reasons detailed in Supplementary Table S3. This yielded 16 new studies eligible for inclusion via the database search. Snowballing of reference lists from included studies and key systematic reviews identified five additional records, two of which satisfied the eligibility criteria. The final review corpus comprised 18 primary studies (Figure 1).

Data from each included study were imported into the NVivo software package (Nvivo v.12.2 (nvivo.software.com/12.2/)) to generate themes and conduct thematic analysis.

### 3.1. Characteristics and Key Findings of Included Studies

A total of 18 studies were included in this review, encompassing data from nine countries across four continents, published between 2015 and 2025. A total of 1631 pediatric patients (the sum of the enrolled sample sizes as reported in each study; Table 2) were included in this review, with individual study sample sizes ranging from 14 participants [16] to 508 participants [17]. Patient age ranged from 0 to 10 years across studies, with most targeting the preschool and early school-age population (3–8 years), consistent with the period of greatest procedural anxiety and limited cooperation. Studies were conducted across nine countries, including those from Asia (India n = 5; South Korea n = 1; Saudi Arabia n = 1; Iran n = 1), North America (USA n = 3), South America (Brazil n = 2), and Africa (Ghana n = 1; South Africa n = 1; Egypt n = 3).

The included studies were mainly randomized controlled trials (RCTs; n = 14, 77.8%), of which six studies [16,18,19,20,21,22] had crossover and eight studies [7,18,22,23,24,25,26,27] had double- or triple-blinded study designs. The remaining studies included three retrospective studies [17,28,29] and one clinical protocol [6]. Patient ages ranged from 0 to 10 years, with the majority of studies (n = 13, 72.2%) focusing on children under eight years of age. The most frequently represented clinical indications were dental anxiety (n = 11, 61.1%) and uncooperative behavior (n = 7, 38.9%) (Table 2).

Intranasal midazolam was the most frequently investigated agent, evaluated either as monotherapy or as a combination component across 11 of 18 studies. Intranasal doses ranged from 0.05 mg/kg [17] to 0.5 mg/kg [26]. Intranasal dexmedetomidine was studied in eight studies at doses of 1–3 µg/kg [16,21,23,25,27,28,29,30], and one further study evaluated buccal dexmedetomidine (2–4 µg/kg) [7]. Intranasal ketamine was assessed in six studies at doses spanning 0.5–10 mg/kg [6,18,23,24,25,30]. Nitrous oxide (N_2_O) served as an adjunct or comparator in four studies [16,20,28,29]. Comparator agents included oral chloral hydrate, oral midazolam with or without hydroxyzine or meperidine, and sublingual formulations.

Mucosal atomization devices (MADs) were the predominant delivery mechanism for intranasal agents, offering consistent droplet size distribution and reliable mucosal absorption [19,20,31]. Aerosolized and atomized preparations were employed in studies by Dubey et al. [25], Sado-Filho et al. [23], and Musani & Chandan [20]. The most frequently employed behavioral scales included the Frankl Behavior Scale (n = 3), Houpt Scale (n = 3), Ohio State University Behavioral Rating Scale (OSUBRS; n = 3), Ramsay Sedation Scale (RSS; n = 2), Venham Anxiety Scale (n = 2) and Pediatric sedation state scale (n = 1).

### 3.2. Thematic Analysis

Thematic analysis of the 18 included studies, conducted in NVivo using the framework described by Braun and Clarke [15], generated six major themes and sixteen subthemes that collectively describe the utilization of intranasal and oral sedation in pediatric dentistry. The themes, subthemes, supporting authors, and representative findings are summarized in Table 3.

Theme 1: Route Efficacy and Behavioral Outcomes

Subtheme 1.1. Intranasal route superior or comparable to alternative routes.

The intranasal route consistently demonstrated high efficacy across multiple drug regimens. Annan et al. reported that the IN ketamine–midazolam combination achieved a 0% failure rate, compared with 30% failure for IN ketamine alone and 20% for IN midazolam alone [24]. Mehran et al. found that IN ketamine was superior to IN midazolam for reducing procedural crying (*p* = 0.002) and movement (*p* = 0.001) during local anesthesia administration [18]. Musani and Chandan demonstrated that IN midazolam at only 0.1 mg/kg combined with nitrous oxide was clinically equivalent to oral midazolam at twice the dose (0.2 mg/kg) [20]. Conversely, Sado-Filho et al. found that IN dexmedetomidine alone and IN dexmedetomidine–ketamine produced equivalent rates of quiet behavior (55.2% vs. 58.4%; *p* = 0.225) [23].

**Table 2:** Characteristics of included studies.

Authors/Year/Country	Study Design	Sample Size/Age Group	Drug/Dosage	Comparator (IN/Oral)	Tools Used to Assess Differences	Dental Procedures Performed	Adverse Events	Conclusion
Annan et al. [24] 2025/Ghana	RCT, double-blind	60 children; ≤2.5 years	Group K: IN ketamine 10 mg/kg	IN ketamine vs. IN midazolam vs. combination	PSSS, separation score (4-point), 4 point separation scale (4-point), hemodynamic monitoring	Computed tomography for oral lesions	Bradycardia 40%, tachycardia 28%, excessive salivation 8%; all transient, no intervention needed	IN combination, the fastest onset (22 ± 9 min), highest sedation success (0% failure).
			Group M: IN midazolam 0.2 mg/kg					
			Group C: IN ketamine 7.5 mg/kg + midazolam 0.1 mg/kg					
Jang et al. [30] 2025/South Korea	RCT, two-center, single-blind	136 enrolled, <7 years	IN DEXKET: IN dexmedetomidine 2 μg/kg + ketamine 3 mg/kg	Oral chloral hydrate 50 mg/kg	PSSS (primary outcome at 15 min), patient acceptance (4-point), separation anxiety (4-point), physician satisfaction (4-point), complication rate	Non-invasive dental procedures.	CH: 16.7% overall complications, 13.6% respiratory	Similar sedation success (75.8% vs. 66.7%, *p* = 0.330); DEXKET significantly lower complications, better patient acceptance; age < 1 yr: fewer complications;
							DEXKET: 3.2% respiratory (*p* = 0.017)	
Janiani et al. [16] 2024/India	RCT, crossover	14 children (42 teeth)/6–8 years	IN dex: 1 μg/kg	IN dexmedetomidine vs. IN midazolam vs. N_2_O	Ramsay Sedation Scale, Houpt Scale, onset time, SpO_2_, pulse rate, safety scale	Anxious children undergoing dental procedures.	Sneezing/coughing: midazolam 64.3%, dex 14.3%,	IN midazolam and N_2_O are equally effective; IN dex inadequate sedation (64.3% agitated), longest onset (36.2 ± 9.47 min); IN midazolam is an effective alternative to N_2_O.
			IN midaz: 0.3 mg/kg					
			N_2_O: 30–50%					
Lalwani et al. [27] 2025/India	RCT, double-blind	48 children/5–8 years	Group I: IN midazolam 0.25 mg/kg	IN midazolam vs. IN dexmedetomidine	Drug acceptance (Likert 5-point), UMSS, FIS anxiety scale, vital parameters	Dental procedures amongst anxious children.	No severe adverse events reported	Dex superior drug acceptance (*p* < 0.001), better effectiveness (*p* < 0.001)
			Group II: IN dexmedetomidine 1.5 μg/kg					
Alhaidari et al. [22] 2024/Saudi Arabia	RCT, double-blind, crossover	32 children (64 visits)/3–6 years	M/F group: Oral midazolam 0.7 mg/kg + IN fentanyl 1 μg/kg	Oral midazolam + IN fentanyl vs. oral midazolam + placebo	MOAA/S sedation scale, behavior scale (4-point), working time, onset time, vital signs	Sedation following local anesthesia for dental extraction.	12.5% overall: hallucinations (6.3%), hiccups (4.7%), vomiting (3.1%), desaturation 92%	M/F significantly improved sedation at parent separation (*p* = 0.032), LA (*p* = 0.018), RD (*p* = 0.035)
			M group: Oral midazolam 0.7 mg/kg + IN placebo					
El-Rouby et al. [7] 2024/Egypt	RCT, parallel, triple-blind	56 children/3–5 years	Group I (DEX-KET): Buccal dexmedetomidine 2 μg/kg + ketamine 2 mg/kg	Buccal DEX-KET vs. buccal DEX alone	OSUBRS behavior scale, drug acceptance (4-point), hemodynamic monitoring, salivary s-IgA, amnesia test, Vernon postoperative questionnaire	Sedation following dental procedures for anxious children.	Minor, comparable between groups; no significant differences (*p* > 0.05)	DEX-KET superior behavior during LA (*p* = 0.017) and treatment (*p* = 0.037); faster recovery (40.2 vs. 55.2 min, *p* < 0.001); DEX alone better drug acceptance (*p* = 0.005)
			Group II (DEX): Buccal dexmedetomidine 4 μg/kg					
Shanmugaavel et al. [32] 2016/India	RCT, parallel	20 children/3–7 years	Group A: IN midazolam 0.2 mg/kg (MAD)	IN vs. sublingual midazolam	Venham’s clinical anxiety scale, salivary cortisol (ELISA), vital signs, Aldrete score	To check reduction in dental anxiety among children following dental procedures.	No adverse effects reported in either group	Both routes are equally effective in reducing anxiety (*p* = 0.004 IN, *p* = 0.003 SL); no significant difference between groups.
			Group B: Sublingual midazolam 0.2 mg/kg (MAD)					
Unkel et al. [28] 2021/USA	Retrospective chart review	149 patients/3–6 years	DEXNO: IN dexmedetomidine 3 μg/kg + N_2_O 65%	IN DEX + N_2_O vs. PO MID + N_2_O vs. PO MID + HYX + N_2_O	Vital signs (BP, HR, SpO_2_, RR, EtCO_2_, ECG), adverse event recording, Aldrete score, PALS criteria	To check reduction in dental anxiety and behavior among children following dental procedures	No major adverse events; DEXNO: 35% hypotension (*p* < 0.001), 1 bradycardia (2%)	IN DEX + N_2_O appears safe, comparable to traditional regimens; DEXNO had significantly more hypotensive events, but all were clinically insignificant and self-correcting
			MIDNO: PO midazolam 0.5–0.7 mg/kg + N_2_O 50–70%					
			MIDHYXNO: PO midazolam 0.5–0.7 mg/kg + PO hydroxyzine 1 mg/kg + N_2_O					
Unkel et al. [29] 2023/USA	Retrospective chart review	146 patients/3–6 years	DEX: IN dexmedetomidine 3 μg/kg + N_2_O	IN DEX + N_2_O vs. PO MID + HYX + N_2_O vs. PO MID + N_2_O	Modified UMSS, AAPD behavior scale, overall effectiveness (completed/effective/ineffective), procedure completion rates, onset time, working time	Effectiveness of sedation following the dental procedures.	Not reported	No statistical difference in effectiveness between regimens (*p* = 0.71); DEX used for more complex procedures. DEX had longest onset (34.8–36.0 min, *p* = 0.003) and longest working time (20.8–26.0 min, *p* < 0.001);
			MIDHYD: PO midazolam 0.5–0.7 mg/kg + PO hydroxyzine 1 mg/kg + N_2_O					
			MID: PO midazolam 0.5–0.7 mg/kg + N_2_O					
Peerbhay & Elsheikhomer [26] 2016/South Africa	RCT, triple-blind	118 children/4–6 years	Group A: IN midazolam 0.5 mg/kg	IN midazolam 0.5 mg/kg vs. 0.3 mg/kg (both via MAD)	Wilson sedation scale, Venham anxiety/behavior scale, Facial Image Scale, pulse oximetry, Aldrete score	Emergency dental procedures.	Burning sensation 9%, sneezing 12%, coughing 4%	100% achieved some sedation; 0.5 mg/kg was more effective for anxiety reduction during LA (*p* = 0.04) and extractions (*p* = 0.03); recovery time was slightly longer
			Group B: IN midazolam 0.3 mg/kg					
Sado-Filho et al. [23] 2021/Brazil	RCT, triple-blind, parallel	88 children/1–7 years	DK group: IN dexmedetomidine 2 μg/kg + IN ketamine	IN DEX + ketamine vs. IN DEX alone	OSUBRS (primary), parental VAS satisfaction, dentist VAS satisfaction, TROOPS adverse events, recovery time	Emergency dental procedures	18.2% overall: desaturation 1.1%, vomiting; nausea + vomiting 4.5%	No difference in quiet behavior (58.4% vs. 55.2%, *p* = 0.225); DK prolonged recovery time 1.3× longer (*p* < 0.05); DEX alone equally efficacious with faster recovery and fewer AEs
			D group: IN dexmedetomidine 2.5 μg/kg alone					
Shaat et al. [21] 2021/Egypt	RCT, crossover	42 children/5–7 years	IN dexmedetomidine 1 μg/kg vs. SL dexmedetomidine 1 μg/kg (same patients, crossover)	IN vs. sublingual dexmedetomidine	Drug acceptance (4-point), onset time, Venham anxiety scale, Vernon postoperative questionnaire, vital signs	Emergency dental procedure amongst anxious children.	No significant adverse events reported.	Sublingual route significantly better acceptance (*p* = 0.01); IN route faster onset (9.87 vs. 13.89 min, *p* < 0.001); both equally effective in preventing anxiety increase during LA (*p* = 0.44)
Gomes et al. [6] 2017/Brazil	RCT (3-arm, parallel, triple-blind)	84 children/2–6 years	Group A (IN): IN ketamine 4 mg/kg + IN midazolam 0.2 mg/kg (via MAD)	IN ketamine + midazolam vs. PO ketamine + midazolam vs. PO midazolam alone	OSUBRS (primary), FLACC, memory test (3-stage), salivary cortisol, World SIVA adverse events tool, VAS (parent/dentist satisfaction), qualitative child interview, cost-effectiveness analysis	Pediatric dental treatment amongst uncooperative children.	Not reported	The rate of combination sedation (IN and PO) was higher compared PO.
			Group B (oral): PO ketamine 4 mg/kg + PO midazolam 0.5 mg/kg					
			Group C (control): PO midazolam 1.0 mg/kg					
Jain et al. [17] 2023/USA	Retrospective, cross-sectional	508 patients/4.5–8.8 years (IQR)	#1: PO MEP 2 mg/kg + PO H 1 mg/kg + PO MID 0.5 mg/kg	Oral MEP-H + IN MID vs. oral MEP-H + oral MID vs. MEP-H alone vs. single MID	Sedation effectiveness (binary), med-to-completion time, med-to-discharge time, teeth completed, HR changes, SpO_2_	Behavior guidance in children during dental treatment.	Oxygen saturation ≥ 96% in all regimens; only 4/504 desaturation events (<96%), all resolved; no serious adverse events	IN MID substitution reduced med-to-discharge time by 23 min vs. PO MID (*p* < 0.05) with comparable efficacy; multi-drug regimens had 2–3.7× higher effectiveness.
			#4: PO MEP 2 mg/kg + oral H 2 mg/kg + IN MID 0.05 mg/kg (via MAD)					
			#2: oral MEP 2 mg/kg + PO H 2 mg/kg					
			#5: Single-agent MID (Oral 0.5 mg/kg or IN 0.25 mg/kg)					
Mehran et al. [18] 2017/Iran	RCT, crossover, double-blind	17 children/3–6 years (mean 4.5 ± 0.9)	IN ketamine 0.5 mg/kg vs. IN midazolam 0.2 mg/kg	IN ketamine vs. IN midazolam	Houpt’s scale (sleep, crying, movement, overall), physiological parameters (HR, BP, RR, SpO_2_), and parental questionnaire	Behavior of 3–6 Year-Old Uncooperative Children in Dental Office.	Ketamine: vomiting 35.3%, nasal discomfort; Midazolam: nasal discomfort 38.2%; no desaturation (<90%)	Ketamine produced significantly less crying (*p* = 0.002), less movement (*p* = 0.001), and more sleepiness (*p* = 0.003) during LA; no significant difference in overall behavior.
Mowafy et al. [19] 2021/Egypt	RCT, crossover	36 children/3–5 years (mean 3.9)	Aerosolized midazolam 0.3 mg/kg via MAD	Buccal vs. intranasal midazolam	Drug acceptance (4-point), onset time, modified Houpt scale (sleep, cry, head resistance, overall behavior)	Behavior management of uncooperative dental patients.	Not systematically reported; buccal is better tolerated; intranasal is associated with burning sensation, sneezing, crying.	Buccal route significantly better acceptance (*p* < 0.001); intranasal faster onset (15.5 vs. 23.0 min, *p* < 0.001); no significant difference in sleep, head resistance, or overall behavior; intranasal had significantly more crying (*p* = 0.01); both routes were safe and effective
Dubey B et al. [25] 2024/India	RCT, triple blinded	47 children/ Age: 3–9 years	Intranasal ketamine 7 mg/kg (atomized)	Intranasal midazolam 0.3 mg/kg (nasal spray) + intranasal dexmedetomidine 3 µg/kg (atomized)	Ohio State Behavioral Rating Scale (OSBRS), University of Michigan Sedation Scale (UMSS), Frankl Behavior Rating Scale	Management of pediatric dental patient for emergency dental treatment.	No adverse event reported	INK (7 mg/kg) demonstrated superior efficacy, safety profile, and patient acceptability. INK showed faster onset, earlier peak sedation, greater depth, shorter recovery, and shorter discharge time.
Musani & Chandan [20], 2015/India	RCT, crossover	30 children/4–10 years	Group O: Oral midazolam 0.2 mg/kg + N_2_O	Oral midazolam + N_2_O vs. IN midazolam + N_2_O	Drug acceptability (4-point), onset time, nasal mask acceptance, Ellis Sedation Score, Houpt scale, safety scale, alertness	Management of uncooperative dental patients.	No adverse effects reported in either group; all safety parameters satisfactory	IN route significantly faster onset (12.1 vs. 20.1 min, *p* < 0.001); faster recovery (higher alertness, *p* < 0.05); IN effective at half the oral dose (0.1 mg/kg vs. 0.2 mg/kg); both combinations safe and well-tolerated
			Group N: IN midazolam 0.1 mg/kg spray + N_2_O					

DB = double-blind; TB = triple-blind; SB = single-blind; IN = intranasal; INK: intranasal ketamine; PO = oral; INMzD = intranasal midazolam + dexmedetomidine DEX = dexmedetomidine; KET = ketamine; MID = midazolam; HYX/H = hydroxyzine; MEP = meperidine; MAD = mucosal atomization device; N_2_O = nitrous oxide; PSSS = Pediatric Sedation State Scale; UMSS = University of Michigan Sedation Scale; OSUBRS = Ohio State University Behavioral Rating Scale; MOAA/S = Modified Observer’s Assessment of Alertness/Sedation; FLACC = Face, Legs, Activity, Cry, Consolability; TROOPS = Tampa Registry of Office-based Procedural Safety; PALS = Pediatric Advanced Life Support; AEs = adverse events; LA = local anesthesia.

**Table 3:** Thematic framework derived from reflexive thematic analysis of 18 included studies.

Theme	Subtheme	Key Studies	Core Finding
1. Route Efficacy and Behavioral Outcomes	1.1 IN superior/comparable to other routes	Annan 2025 [24]; Mehran 2017 [18]; Musani 2015 [20]; Sado-Filho 2021 [23]	IN combination (ketamine + midazolam): 0% failure; IN effective at half the oral midazolam dose
	1.2 Multi-drug regimens may be more effective	Jain 2023 [17]; Alhaidari 2024 [22]; El-Rouby 2024 [7]; Dubey 2024 [25]	Multi-drug: 2.65–3.73 higher efficacy; fentanyl addition improves three sedation endpoints
	1.3 IN effective in specific populations	Jang 2025 [30]; Unkel 2021 [28]; Unkel 2023 [29]	IN DEXKET: 79.2% success in ages 1–7 yr and 51.6% for chloral hydrate; preferred for complex cases
2. Onset of Action	2.1 IN route fastest onset	Shaat 2021 [21]; Mowafy 2021 [19]; Musani 2015 [20]; Annan 2025 [24]; Dubey 2024 [25]	IN onset: 9.87–22 min in same-drug comparisons; sublingual/buccal/oral consistently slower by 4–8 min
	2.2 Slower onset: oral/buccal/sublingual routes and IN dexmedetomidine	Lalwani 2025 [27]; Shanmugaavel 2016 [32]	Sublingual midazolam slower than IN midazolam; within the IN route, dexmedetomidine slowest (41.42 min vs. 30.79 min for IN midazolam)
3. Recovery and Discharge	3.1 IN midazolam = fastest recovery	Peerbhay 2016 [26]; Annan 2025 [24]; Jain 2023 [17]; Musani 2015 [20]	IN midazolam recovery 16.5–18.8 min; discharge 23 min earlier vs. oral midazolam
	3.2 Dexmedetomidine/combinations prolong recovery	Sado-Filho 2021 [23]; Unkel 2021 [28]	DEX-KET: 1.3× longer recovery than DEX alone; DEX working time 23 min vs. oral regimens
4. Patient Acceptance and Tolerability	4.1 Buccal/sublingual > IN acceptance	Mowafy 2021 [19]; Shaat 2021 [21]; Shanmugaavel 2016 [32]; Lalwani 2025 [27]	Buccal/sublingual significantly better accepted (*p* < 0.001); IN dex better accepted than IN midazolam
	4.2 MAD device improves IN tolerability	Peerbhay 2016 [26]; Janiani 2024 [16]; Alhaidari 2024 [22]	Lidocaine pre-treatment reduced burning 43%→9%; IN dex: 14.3% mucosal events vs. 64.3% for IN midazolam
5. Adverse Events and Safety	5.1 DEX: hypotension, bradycardia	Unkel 2021 [28]; Sado-Filho 2021 [23]	DEXNO: 35% hypotension (all self-correcting); DEX alone AE rate 15% vs. 20% for DEX-KET
	5.2 Ketamine: vomiting, CV stimulation	Mehran 2017 [18]; Annan 2025 [24]; Sado-Filho 2021 [23]	Ketamine vomiting 35.3%; bradycardia 40%/tachycardia 28% (all transient); no desaturation < 90%
	5.3 Midazolam: local irritation, minor respiratory	Janiani 2024 [16]; Peerbhay 2016 [26]; Alhaidari 2024 [22]	Sneezing/coughing 64.3%; one SpO_2_ 94% event; one desaturation to 90% (self-resolved within 3 min)
	5.4 Risk factors for AEs	Jang 2025 [30]	Age < 1 yr: DEXKET 2.6% vs. chloral hydrate 22.9% complications (*p* = 0.012)
6. Special Populations	6.1 SHCN children	Jang 2025 [30]; Unkel 2021 [28]; Gomes 2017 [6]	IN dexmedetomidine preferred for complex/SHCN presentations; no ICU admissions across all studies
	6.2 Infants < 1 year	Jang 2025 [30]; Sado-Filho 2021 [23]	IN DEXKET: 2.6% complications in infants vs. 22.9% for oral chloral hydrate
	6.3 Cardiac patients	Jang 2025 [30]; Jain 2023 [17]	IN DEXKET safe in 80% cardiac cohort; multi-drug: 2.65–3.73× higher efficacy without hemodynamic compromise

IN = intranasal; DEX = dexmedetomidine; KET/DEXKET = dexmedetomidine + ketamine; DEXNO = dexmedetomidine + nitrous oxide; MAD = mucosal atomization device; AE = adverse event; SHCN = special healthcare needs children; CV = cardiovascular.

Subtheme 1.2. Multi-drug regimens may be more effective than monotherapy.

Jain et al. reported that multi-drug regimens comprising meperidine, hydroxyzine, and midazolam had 2.65 to 3.73 times higher odds of achieving effective sedation compared with single-agent midazolam (*p* < 0.05) [17]. Alhaidari et al. demonstrated that the addition of IN fentanyl (1 μg/kg) to oral midazolam (0.7 mg/kg) significantly improved sedation at parental separation (*p* = 0.032), during local anesthesia (*p* = 0.018), and throughout the restorative procedure (*p* = 0.035) compared with oral midazolam plus intranasal placebo [22]. Al-Rouby et al. reported that buccal dexmedetomidine–ketamine produced superior child behavior during local anesthesia (*p* = 0.017) and operative treatment (*p* = 0.037), together with a significantly shorter sedation duration (40.2 vs. 55.2 min; *p* < 0.001) [7]. However, these findings contrast with the equivalence reported by Sado-Filho et al., who found no significant advantage from adding ketamine to intranasal dexmedetomidine [23].

Subtheme 1.3. Intranasal sedation is effective in specific pediatric populations.

Jang et al. conducted a two-center RCT in children undergoing diagnostic cardiac catheterization and dental procedures, demonstrating that IN dexmedetomidine–ketamine achieved significantly higher sedation success in children aged 1–7 years (79.2% vs. 51.6%) and a substantially lower overall complication rate (3.2% vs. 16.7%) compared with oral chloral hydrate (*p* = 0.017) [30]. In the two included studies by Unkel et al., it was confirmed that IN dexmedetomidine combined with nitrous oxide was comparably effective to oral dosage for pediatric dental sedation in community practice settings [28,29].

Theme 2: Onset of Action
Subtheme 2.1. The intranasal route provides the fastest onset of sedation.

Shaat et al. reported an onset of 9.87 min for IN dexmedetomidine versus 13.89 min for the sublingual route (*p* < 0.001) [21]. Mowafy et al. documented IN midazolam onset at 15.5 min compared with 23.0 min via the buccal route (*p* < 0.001) [19]. Musani and Chandan recorded IN midazolam onset at 12.1 min versus 20.1 min for oral midazolam (*p* < 0.001) [20]. The combination of IN ketamine and midazolam achieved the fastest recorded onset overall (22 ± 9 min), outperforming either agent alone [24]. The physiological basis for this advantage, as elaborated by Peerbhay and Elsheikhomer, lies in direct drug absorption through the cribriform plate into cerebrospinal fluid and brain tissue, thereby bypassing first-pass hepatic metabolism and the blood–brain barrier [26].

Subtheme 2.2. Onset is slower via oral, buccal, and sublingual routes and with intranasal dexmedetomidine.

Lalwani et al. found that IN dexmedetomidine had a markedly longer time to clinical effect (41.42 min) than IN midazolam (30.79 min) [27]. Shanmugaavel et al. confirmed that intranasal midazolam trended toward faster onset than sublingual midazolam, consistent with the relatively greater absorptive vascularity of the nasal mucosa [32]. These differences carry direct clinical implications for procedure scheduling and pre-sedation timing.

Theme 3: Recovery and Discharge Times
Subtheme 3.1. Intranasal midazolam is associated with faster recovery.

Peerbhay and Elsheikhomer documented mean recovery times of 16.5 min for the 0.3 mg/kg dose and 18.8 min for the 0.5 mg/kg dose (*p* = 0.03) [26]. Annan et al. found that IN midazolam alone produced the fastest recovery (63 ± 25 min) and earliest discharge (92 ± 29 min) compared with ketamine-containing regimens (*p* = 0.007 and *p* = 0.036, respectively) [24]. Musani and Chandan reported significantly higher post-procedural alertness with IN midazolam compared with oral midazolam (*p* < 0.05) [20]. Jain et al. confirmed these findings, showing that substituting IN midazolam for oral midazolam within a multi-drug regimen reduced median time to discharge by 23 min (*p* < 0.05) [17].

Subtheme 3.2. Dexmedetomidine-containing and combination regimens prolong recovery.

Sado-Filho et al. reported that adding ketamine to IN dexmedetomidine extended recovery time 1.3-fold compared with dexmedetomidine alone (*p* < 0.05) [23]. Unkel et al. demonstrated that the IN dexmedetomidine group had the longest onset (34.8–36.0 min; *p* = 0.003) and longest working time (20.8–26.0 min; *p* < 0.001) compared with oral regimens [29].

Theme 4: Patient Acceptance and Tolerability
Subtheme 4.1. Buccal and sublingual routes demonstrate superior patient acceptance.

Mowafy et al. documented significantly higher acceptance for buccal midazolam compared with intranasal administration (*p* < 0.001) [19]. Shaat et al. similarly reported better acceptance for sublingual dexmedetomidine relative to the intranasal route (*p* = 0.01) [21]. Shanmugaavel et al. confirmed that sublingual midazolam produced better acceptance, with intranasal delivery causing burning, irritation, and lacrimation [32]. Conversely, Lalwani et al. found that IN dexmedetomidine was significantly better accepted than IN midazolam (*p* < 0.001) [27].

Subtheme 4.2. The mucosal atomization device improves intranasal tolerability.

A mucosal atomization device (MAD) was employed in 50% of included studies (n = 9) to optimize drug dispersion and reduce local irritation. Peerbhay and Elsheikhomer demonstrated that lidocaine pre-treatment reduced the incidence of burning sensation from 43% to 9% and recommended concentrated drug formulations (25–40 mg/mL) to minimize volume-related discomfort [26]. Janiani et al. reported that coughing or sneezing occurred in 64.3% of children receiving IN midazolam versus only 14.3% with IN dexmedetomidine via MAD, reinforcing that drug choice significantly influences mucosal tolerability [16].

Theme 5: Adverse Events and Safety Profile
Subtheme 5.1. Dexmedetomidine is associated with hypotension and bradycardia

Unkel et al. documented hypotension in 35% of children receiving IN dexmedetomidine with nitrous oxide (*p* < 0.001), alongside bradycardia in 2% [28]. However, all events were clinically insignificant and self-correcting, requiring no pharmacological intervention. Among the studies evaluating IN dexmedetomidine, Sado-Filho et al. (2021) observed a lower total adverse event rate with dexmedetomidine alone (15%) than with dexmedetomidine–ketamine (20%) [23]. Alhaidari et al. reported a 12.5% overall adverse event rate with IN fentanyl combined with oral midazolam, comprising hallucinations (6.3%), hiccups (4.7%), and vomiting (3.1%), all of which were self-limiting and required no pharmacological reversal [22].

Subtheme 5.2. Ketamine is associated with vomiting and cardiovascular stimulation.

Mehran et al. reported postoperative vomiting in 35.3% of children receiving IN ketamine, compared with nasal discomfort in 38.2% of those receiving IN midazolam [18]. Annan et al. documented bradycardia (40%), tachycardia (28%), and excessive salivation (8%) across ketamine-containing groups, noting that all events were transient and resolved without clinical intervention [24]. Sado-Filho et al. observed a combined nausea and vomiting rate of 4.5% and desaturation in 1.1% of children receiving IN dexmedetomidine–ketamine [23].

Subtheme 5.3. Midazolam is associated with minor respiratory and local irritant effects.

Janiani et al. reported a transient oxygen desaturation (SpO_2_ 94%) in one patient receiving IN midazolam and coughing or sneezing in 64.3% of participants [16]. Peerbhay and Elsheikhomer documented one episode of desaturation to 90% that was resolved spontaneously within three minutes, with no serious adverse events identified across 118 participants [26]. Burning sensation was reported in 9% and sneezing in 12% of participants in that cohort.

Subtheme 5.4. Patient-level risk factors for adverse events

Jang et al. identified patient age as a significant modulator of sedation safety: children under one year of age experienced fewer complications with IN dexmedetomidine–ketamine (2.6%) compared with oral chloral hydrate (22.9%; *p* = 0.012) [30]. Across all 18 included studies, no deaths, anaphylactic reactions, need for unplanned airway interventions, or admissions to intensive care were reported. The collective safety evidence supports the conclusion that, with appropriate patient selection, monitoring, and drug dose adjustment, IN and oral sedation combinations appeared to be well tolerated in the included studies, with no serious adverse events reported.

Theme 6: Special Pediatric Populations
Subtheme 6.1. Children with special healthcare needs.

Seven studies (38.9%) included or discussed children with special healthcare needs (SHCN), encompassing autism spectrum disorder (ASD), congenital heart disease, intellectual disabilities, and metabolic disorders. Jang et al. specifically enrolled children with cardiac conditions (80% of the cohort) and demonstrated that IN dexmedetomidine–ketamine could be used safely without clinically significant hemodynamic compromise, despite dexmedetomidine’s negative chronotropic properties [30]. Unkel et al. noted that IN dexmedetomidine was preferentially selected by clinicians for children with more complex behavioral or medical presentations [28,29].

Subtheme 6.2. Infants under one year of age.

Jang et al. provided the only included study to specifically analyze outcomes in infants under one year, demonstrating that IN dexmedetomidine–ketamine produced a complication rate of 2.6% in this sub-group compared with 22.9% for oral chloral hydrate (*p* = 0.012) [30]. Sado-Filho et al. enrolled children as young as one year of age in their RCT, reporting a desaturation rate of 1.1% and a combined nausea-and-vomiting rate of 4.5%, establishing that IN sedation with dexmedetomidine ± ketamine carries an acceptable safety margin even in the youngest dental patients [23].

Subtheme 6.3. Children with cardiac conditions.

The inclusion of cardiac patients in Jang et al. offered direct evidence that IN dexmedetomidine–ketamine does not precipitate clinically significant bradycardia or hemodynamic instability in pediatric cardiac patients [30]. Jain et al. reported that multi-drug combinations including IN midazolam were associated with a 2.65 to 3.73 times higher probability of effective sedation compared with single-agent midazolam alone [17].

### 3.3. Evidence Gaps

Systematic mapping of the included studies revealed several gaps in the evidence base (Table 4). Most fundamentally, there is no consensus definition of sedation success across the literature: studies define success as completion of the dental procedure, achievement of a target sedation score, absence of procedure interruptions, or a minimum proportion of time during which the child had supportive behavior. This heterogeneity renders cross-study comparison unreliable and substantially hampers meta-analytic synthesis. The cost-effective analyses and implementation research are entirely absent from the published literature, creating a significant barrier to policy development and equitable clinical translation.

**Figure 2:**
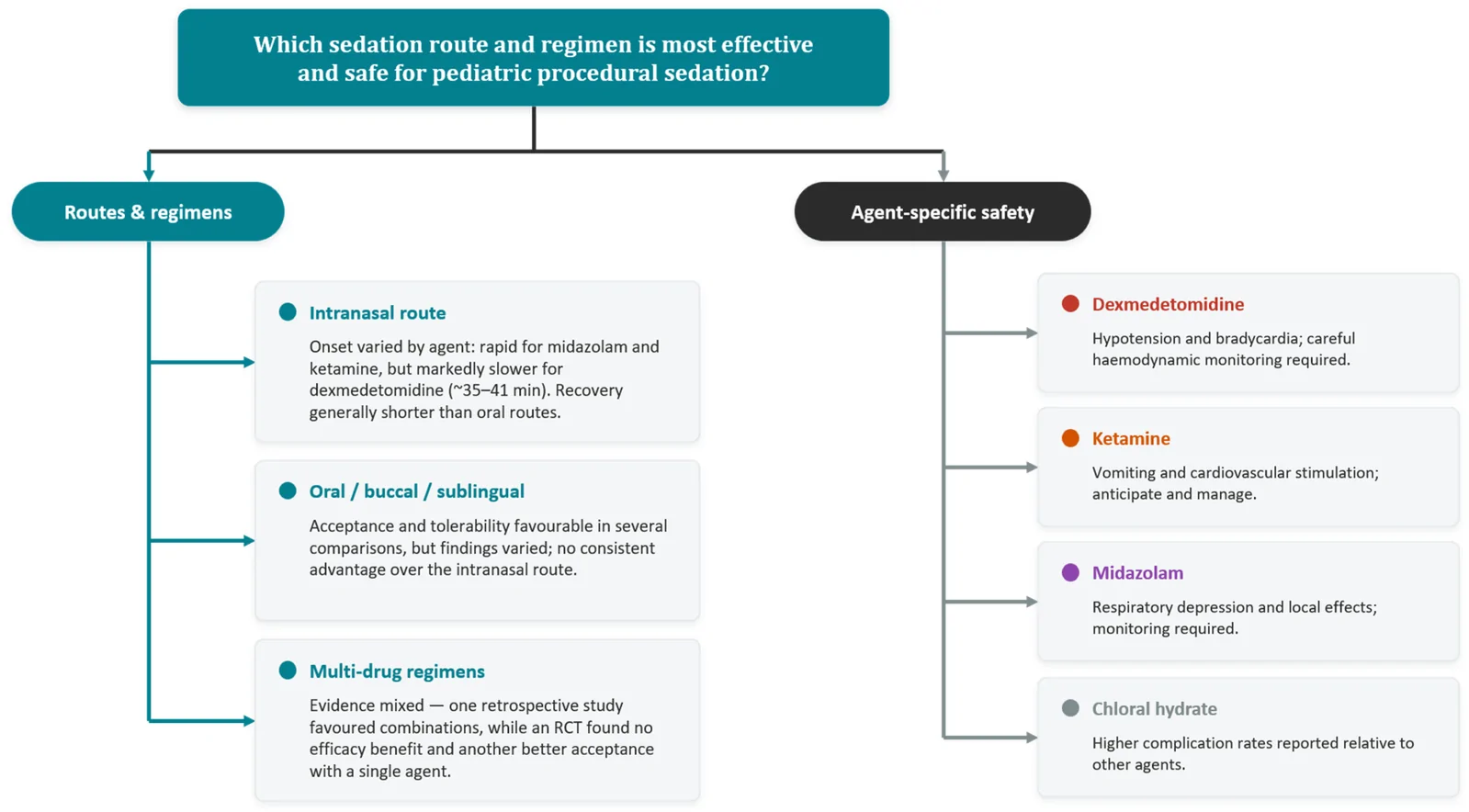
Framework illustrating the comparative clinical utility of intranasal and oral sedation routes and drug-specific adverse event profiles in pediatric dental sedation, derived from thematic analysis of included studies (figure made with BioRender software [https://app.biorender.com/]).

Figure 2 demonstrates a reflective framework for selecting optimal sedation routes and pharmacological regimens in pediatric procedural sedation. It illustrates that onset with the intranasal route varied by agent—rapid for midazolam and ketamine but markedly slower for dexmedetomidine—while recovery was generally shorter than with oral routes; that oral, buccal, and sublingual routes showed favorable acceptance and tolerability in several comparisons, although findings varied and no consistent advantage over the intranasal route emerged; and that evidence on multi-drug versus single-agent regimens was mixed, with one retrospective study favoring combinations, while an RCT found no added efficacy benefit and another study reported better acceptance with a single agent. Concurrently, the figure highlights comparative drug-specific adverse effect profiles, thereby supporting individualized, evidence-based selection of intranasal–oral combination protocols in pediatric dentistry.

## 4. Discussion

The present scoping review systematically mapped and thematically analyzed the published evidence on intranasal (IN) and oral (PO) pharmacological sedation combinations used for procedural sedation in pediatric dentistry. Across 18 studies including 1580 children, six major themes and 16 subthemes emerged. These themes mainly evaluated the clinical efficacy of combination IN and oral sedation. Studies support the utilization of combination drugs (IN and PO) for procedural sedation, suggesting the requirement for proper dosage regimen and clinical protocol.

Studies have supported the utilization of combination drug in controlling anxiety and behavior in children. Jain et al. reported that multi-drug combinations had 2.65–3.73 times higher odds of effective sedation compared with only oral or intranasal sedation [17]. Gao and Wu reported that the combination of drugs increases its efficacy if used in recommended dosage [8]. However, Sado-Filho et al. found no significant difference in quiet behavior between IN dexmedetomidine–ketamine and IN dexmedetomidine alone (58.4% vs. 55.2%; *p* = 0.225) [23]. The finding implies that when a primary agent is used at a clinically adequate dose, supplemental agents may add adverse effect risk without behavioral benefit [23]. Al-Rouby et al. found that buccal dexmedetomidine alone has higher acceptance over dexmedetomidine–ketamine, despite the combination achieving better procedural outcomes [7]. Overall, the evidence on multi-drug versus single-agent regimens is heterogeneous and design-dependent, and any inference of superiority for combination regimens is limited by the small number of studies and their differing designs and outcome definitions.

Musani and Chandan reported that IN midazolam 0.1 mg/kg combined with nitrous oxide have similar effect as oral midazolam at double the dose (0.2 mg/kg) [20]. Peerbhay and Elsheikhomer reported that drug given via IN directly absorb in cribriform plate, bypassing first-pass metabolism [26]. This pharmacokinetic advantage of the IN route has been confirmed in bioavailability studies reporting intranasal midazolam F-values of 50–75% compared with 40–50% for oral administration [26].

Studies have reported that intranasal drugs offer a faster onset of action than the sublingual route [31,33,34]. Shaat et al. reported no significant difference in children’s behavior with the combination drug and oral administration; however, during the procedure, the onset of the sublingual drug was higher than the intranasal drug [21]. Musani et al. reported the quick effect of intranasal spray in anxious children [20]. The physiological basis of faster absorption of intranasal drugs could be due to direct absorption of the drug through cribriform plates into the cerebrospinal fluid and brain tissues [35,36]. Studies have reported that intranasal ketamine had a mean sedation score of 4, which is clinically acceptable in children below 7 years [1,37]. This rapid onset has a clinical advantage in a pediatric dental setting, as it reduces the child’s waiting and anxiety period and improves the efficiency of the clinical workflow [38]. The slower onset of oral and even buccal routes, while still effective, requires more foresight in clinical scheduling and may be less suitable for managing acute anxiety at the moment of treatment [13].

Dexmedetomidine has a significantly longer recovery duration compared to both IN midazolam and IN ketamine [3]. This finding is consistent with previously published meta-analysis reporting that IN dexmedetomidine had a prolonged recovery time [9,39]. Included studies reported that IN midazolam had a faster recovery profile with a mean difference of 19.1 min (*p* = 0.05). Preethy et al. reported that ketamine had a faster recovery time, reporting that the given drug provides dissociative sedation and rapid clearance [40]. Sado-Filho et al. reported extended recovery times for dexmedetomidine–ketamine combinations [23]. Al-Rouby et al. reported that DEX-KET had a shorter duration than DEX alone [7]. This could be attributed to differences in dosing, the specific patient population, or the definition of “sedation duration”.

Various satisfaction and behavioral scales were used to monitor patients’ acceptance and tolerance of IN in combination with oral sedative drugs. Studies reported that parental satisfaction was highest with dexmedetomidine, which provides smoother sedation [16,41]. In a meta-analysis, it was reported that parental satisfaction was significantly higher with dexmedetomidine compared to midazolam, despite its association with prolonged sedation [42]. IN midazolam was accepted in patients requiring faster sedation. IN midazolam demonstrated higher variability in patients’ acceptance, mostly because of its reduced adverse events. However, studies have reported burning sensation, irritation, and sneezing associated with IN midazolam, increasing anxiety among the patients during administration [18,27,43]. Ketamine was generally well accepted in patients due to its anxiolytic effects. However, some studies reported adverse events like hypersalivation and postoperative agitation with ketamine [16,30,44]. These findings underscore the need for individualized sedation strategies according to the patients’ needs and procedural requirements.

The use of a mucosal atomization device (MAD) was standard in many studies to improve drug dispersion and absorption. Peerbhay and Elsheikhomer successfully demonstrated that lidocaine pre-treatment and the use of concentrated drug formulations significantly reduced the burning sensation [26]. Furthermore, drug selection also plays a significant role in patients’ acceptance and tolerance. This theme underscores that choosing a sedation route is not just about pharmacological effect but also about the child’s immediate experience.

The most common adverse event reported was nasal irritation and prolonged sedation, requiring constant monitoring. Studies have reported that dexmedetomidine was consistently associated with hypotension and bradycardia [28,29,31]. Liu et al. reported that these complications require careful patient selection and vigilant monitoring [44]. The most frequent complication associated with ketamine was vomiting and tachycardia [27,41]. Midazolam, while generally safe, was linked to minor respiratory events like desaturation and local irritating effects [16]. However, the current review opposes the use of chloral hydrate, as this drug has significantly higher complications compared to combinations like IN dexmedetomidine–ketamine [30]. The findings from the given theme are consistent with the American Academy of Pediatric Dentistry’s (AAPD’s) sedation guidelines, which emphasize the importance of post-procedural monitoring [13].

A novel and significant contribution of the current scoping review is the inclusion of special populations and evidence supporting the requirement of sedation in this population. Jang et al. provided evidence for the safe use of IN dexmedetomidine–ketamine in infants under one year of age [30]. Studies have reported that sedation is effective in children with intellectual disability [3,28,38]. However, in the study by Hossenally and Doughty, it was reported that in patients with autism spectrum disorder (ASD), quick administration of IN midazolam at the second visit is effective [45]. In the study by Schulz-Weidner et al., it was reported that a larger dose of propofol is better for achieving sedation in patients with intellectual impairments [46]. However, there are limited studies supporting the combination of IN and oral sedative drugs in children with special needs, which may be attributed to the aggressive behavior of children with special needs.

The difference in monitoring on children under conscious sedation was reported in the included studies. Jang et al. monitored infants under sedation using a six-point Pediatric Sedation State Scale (PSSS) [30]. Schulz-Weidner et al. in their retrospective analysis only measured the adverse events recorded amongst intellectually impaired children undergoing dental treatment following conscious sedation [46]. Liu et al. evaluated level of sedation following code system [44]. These variations in monitoring can be attributed to the inclusion of the patients.

Safety is possibly the most significant outcome in pediatric dental sedation; however, the current synthesis reported a substantial heterogeneity in how adverse events were defined and reported across the included studies. Respiratory problems were the most frequently reported adverse events, but the thresholds used to define desaturation varied, and several studies reported events without any explicit definition, precluding meaningful pooling. Similarly, cardiovascular events, paradoxical reactions, and recovery-phase outcomes such as prolonged recovery and delayed discharge were reported inconsistently, and standardized, validated discharge criteria were applied in few studies. These limitations mean that the pooled or narrative event frequencies reported should be interpreted as conservative estimates that are as likely to reflect reporting and detection practices as true underlying risk. Because pediatric patients are especially vulnerable to airway compromise and recovery-phase complications, this lack of standardization is not an insignificant methodological concern but a direct barrier to establishing the comparative safety of sedation regimens. Future studies should adopt standardized definitions for adverse events and reporting frameworks for procedural sedation, report monitoring modalities and discharge criteria clearly, and pre-specify safety outcomes in similar ways so that future syntheses can provide a reliable quantitative comparison.

### Strengths and Limitations

The dual-analytic methodology combining quantitative evidence mapping with reflexive thematic analysis in NVivo represents a methodological strength. By analyzing the qualitative and interpretive content of the included studies, the thematic approach was able to identify patterns of clinical reasoning, parental perspective, and implementation challenge. Pre-registration on the Open Science Framework and adherence to PRISMA-ScR reporting guidelines further support the review’s transparency and reproducibility.

Despite the current scoping review discussing different tools and safety measures used to check the efficacy of IN and oral combinations of sedation, consistent with scoping review methodology, which aims to map evidence rather than appraise its quality, it did not include a formal quality assessment for the included studies. During the development of themes, the authors mainly focused on outcomes like efficacy, patients’ acceptance, recovery period, safety measures, and special need population, but did not study dentist-reported outcomes and protocols followed during the administration. This could have altered the findings of the current review. The review has not focused on contraindications of IN sedations, which should be considered in future research.

## 5. Conclusions

Within the limitations of the current scoping review, it could be concluded that combination intranasal and oral sedation may represent an effective and clinically adaptable approach for pediatric dental sedation, with no serious adverse events reported among the included studies. In some studies, combination intranasal and oral sedation was associated with faster recovery than monotherapy. The six themes and sixteen subthemes developed in the given review provided an in-depth analysis of patient-reported outcomes like recovery time, patient acceptance, safety, and measures taken to manage children with special needs. However, the observed variations in the recovery period, patients’ acceptance, and adverse events highlight the need for personalized sedation strategies. Because no formal risk-of-bias or quality appraisal was undertaken, and the included studies were predominantly small, single-center, and often unblinded, these conclusions should be regarded as hypothesis-generating rather than practice-guiding.

## Figures and Tables

**Table 1:** PCC framework and concept blocks.

PCC Element	Concept Block	Representative Terms
Population	Children and adolescents (0–18 years)	child, children, infant, toddler, preschool, paediatric/pediatric, adolescent, teenager, youth, juvenile, schoolchild, minors
Concept	Sedation terminology	conscious sedation, minimal/moderate/deep sedation, procedural sedation, dental sedation, sedative, anxiolysis, premedication, pharmacological behaviour management/guidance
Concept	Named sedative agents	midazolam, dexmedetomidine, ketamine, chloral hydrate, hydroxyzine, promethazine, diazepam, triclofos, melatonin, clonidine, pethidine/meperidine, sufentanil, fentanyl, zolpidem, buspirone, propofol, benzodiazepines
Concept	Route of administration	intranasal, intra-nasal, transnasal, nasal, atomised/atomized, mucosal atomisation device, oral administration, orally administered, per os, peroral, by mouth, sublingual, buccal, transmucosal
Context	Dental care	dentistry, paediatric dentistry, dental care, dental treatment/procedure, dental anxiety, oral surgery, tooth extraction, exodontia, pulpotomy, pulpectomy, caries, oral rehabilitation

**Table 4:** Key evidence gaps by domain.

Domain	Primary Gap	Consequence	Priority Level
Outcome measurement	No standardized sedation success definition	Cross-trial comparison unreliable; meta-analysis not feasible	Critical
Study design	Small (median n = 52), single-center, often unblinded	Limited generalizability; high risk of bias	High
Populations	Infants < 1 yr, SHCN, neurodiverse children understudied	Evidence not representative of clinical burden	High
Dosing	Optimal IN DEX:KET ratio undefined; IN fentanyl understudied	Suboptimal dosing in practice	Medium
Safety	Risk predictors poorly characterized	Cannot pre-identify high-risk patients	High
Health economics	No cost-effectiveness analyses published	Cannot inform policy or commissioning	Medium
Implementation	No dissemination or implementation research	Evidence not translated to clinical practice	Medium
Long-term outcomes	No follow-up beyond 24 h post-procedure	Unknown lasting behavioral or physiological impact	Medium

## Data Availability

Data will be available on request from corresponding author.

## Supplementary Materials

The following supporting information can be downloaded at: https://www.mdpi.com/article/10.3390/children13091153/s1; Table S1: PRISMA-ScR checklist; Table S2: Search strategy; Table S3: Excluded studies with reasons.

## References

[1] Clinical Affairs Committee-Behavior Management Subcommittee, American Academy of Pediatric Dentistry (2015). Guideline on Behavior Guidance for the Pediatric Dental Patient. Pediatr. Dent..

[2] Almutawwif M., Gogandi H., Faqiri F., Aloufi A., Bamunif M., Hazzazi A., Abed H. (2025). Evaluating the Efficacy of Intranasal Sedation in the Dental Setting: A Scoping Review. Spec. Care Dent..

[3] Berry E.J. (2025). A review on moderate sedation in pediatric dentistry: A focus on indications, safety and the newest medications. J. Oral Maxillofac. Anesth..

[4] Gentz R., Casamassimo P., Amini H., Claman D., Smiley M. (2017). Safety and Efficacy of 3 Pediatric Midazolam Moderate Sedation Regimens. Anesth. Prog..

[5] Gharavi M., Salem K., Ahmadi S.A.Y., Baradaran H.R. (2025). A Comparative Evaluation of Midazolam, Ketamine and Their Combination as Sedative Agents in Pediatric Dentistry: A Systematic Review and Meta-analysis. Front. Dent..

[6] Gomes H.S., Miranda A.R., Viana K.A., Batista A.C., Costa P.S., Daher A., Machado G.C., Sado-Filho J., Vieira L.A., Corrêa-Faria P. (2017). Intranasal sedation using ketamine and midazolam for pediatric dental treatment (NASO): Study protocol for a randomized controlled trial. Trials.

[7] El-Rouby S.H., Crystal Y.O., Elshafie A.M., Wahba N.A., El-Tekeya M.M. (2024). The effect of dexmedetomidine-ketamine combination versus dexmedetomidine on behavior of uncooperative pediatric dental patients: A randomized controlled clinical trial. J. Appl. Oral Sci..

[8] Gao F., Wu Y. (2023). Procedural sedation in pediatric dentistry: A narrative review. Front. Med..

[9] Romagnolo F.U., Bottesini V.C., De Souza R.C.C., Duarte D.A. (2025). Adverse Events of Procedural Sedation in Pediatric Dentistry: Scoping Review. Cumhur. Dent. J..

[10] Inchingolo F., Inchingolo A., Ferrante L., De Ruvo E., Di Noia A., Palermo A., Inchingolo A., Dipalma G. (2024). Pharmacological sedation in paediatric dentistry. Eur. J. Paediatr. Dent..

[11] Heard C., Smith J., Creighton P., Joshi P., Feldman D., Lerman J. (2010). A comparison of four sedation techniques for pediatric dental surgery. Paediatr. Anaesth..

[12] Oza R.R., Sharma V., Suryawanshi T., Lulla S., Bajaj P., Dhadse P. (2022). Comparative analysis of sedative efficacy of dexmedetomidine and midazolam in pediatric dental practice: A systematic review and meta-analysis. Cureus.

[13] Ashley P., Anand P., Andersson K. (2021). Best clinical practice guidance for conscious sedation of children undergoing dental treatment: An EAPD policy document. Eur. Arch. Paediatr. Dent..

[14] Moher D., Shamseer L., Clarke M., Ghersi D., Liberati A., Petticrew M., Shekelle P., Stewart L.A., Group P.-P. (2015). Preferred reporting items for systematic review and meta-analysis protocols (PRISMA-P) 2015 statement. Syst. Rev..

[15] Braun V., Clarke V. (2006). Using thematic analysis in psychology. Qual. Res. Psychol..

[16] Janiani P., Gurunathan D., Manohar R. (2024). Comparative evaluation of intranasal dexmedetomidine, intranasal midazolam, and nitrous oxide for conscious sedation of anxious children undergoing dental treatment: A randomized cross-over trial. J. Indian Soc. Pedod. Prev. Dent..

[17] Jain G., Curran R., Jones R.S. (2023). Retrospective Evaluation of Moderate Sedation Visits That Used Oral Meperidine and Hydroxyzine With Oral or Intranasal Midazolam. J. Dent. Child..

[18] Mehran M., Tavassoli-Hojjati S., Ameli N., Zeinabadi M.S. (2017). Effect of Intranasal Sedation Using Ketamine and Midazolam on Behavior of 3-6 Year-Old Uncooperative Children in Dental Office: A Clinical Trial. J. Dent..

[19] Mowafy Y.N., Wahba N.A., Gho Neim T.M., Mahmoud G.M. (2021). Efficacy of buccal versus intranasal route of administration of midazolam spray in behavior management of preschool dental patients. Quintessence Int..

[20] Musani I.E., Chandan N.V. (2015). A comparison of the sedative effect of oral versus nasal midazolam combined with nitrous oxide in uncooperative children. Eur. Arch. Paediatr. Dent..

[21] Shaat M.A., Bakry N.S., Elshafie A.M., Talaat D.M. (2022). Intranasal versus sublingual route of dexmedetomidine sedation in paediatric dentistry: A randomized controlled clinical trial. Int. J. Paediatr. Dent..

[22] Alhaidari R.I., AlSarheed M., Sheta S.A., Aldhubaiban M. (2022). Intranasal Fentanyl Combined with Oral Midazolam for Pediatric Dental Sedation: A Controlled Randomized Blinded Crossover Clinical Trial. Pediatr. Dent..

[23] Sado-Filho J., Corrêa-Faria P., Viana K.A., Mendes F.M., Mason K.P., Costa L.R., Costa P.S. (2021). Intranasal Dexmedetomidine Compared to a Combination of Intranasal Dexmedetomidine with Ketamine for Sedation of Children Requiring Dental Treatment: A Randomized Clinical Trial. J. Clin. Med..

[24] Annan B.N.Y., Owusu Darkwa E., Anno A., Vanderpuye N.M., Baffour-Awuah L., Obeng Adjei G.-I., Danso O.-S., Essuman R., Aryee G., Djagbletey R. (2025). Appropriateness and safety of using the intranasal route for sedation during pediatric computed tomography: A randomized controlled trial at Korle-Bu teaching hospital. J. Int. Med. Res..

[25] Dubey B., Singh N., Kumar S. (2024). Comparison of intranasal ketamine with intranasal midazolam and dexmedetomidine combination in pediatric dental patients for procedural sedation: A crossover study. J. Indian Soc. Pedod. Prev. Dent..

[26] Peerbhay F., Elsheikhomer A.M. (2016). Intranasal Midazolam Sedation in a Pediatric Emergency Dental Clinic. Anesth. Prog..

[27] Lalwani Y., Dave B., Shah L. (2025). Comparative evaluation of the effectiveness and acceptance of intranasal dexmedetomidine and intranasal midazolam for sedation in children aged 5–8 years using a mucosal atomizer device: A randomized controlled clinical study. J. Dent. Anesth. Pain. Med..

[28] Unkel J.H., Berry E.J., Ko B.L., Amarteifio V., Piscitelli W., Reinhartz D., Reinhartz J., Warren R. (2021). Effectiveness of Intranasal Dexmedetomidine with Nitrous Oxide Compared to Other Pediatric Dental Sedation Drug Regimens. Pediatr. Dent..

[29] Unkel J.H., Cruise C., Rice A., Macdonald J., Berry E.J., Reinhartz J., Reinhartz D. (2021). A Retrospective Evaluation of the Safety Profile of Dexmedetomidine and Nitrous Oxide for Pediatric Dental Sedation. Pediatr. Dent..

[30] Jang Y.-E., Joo E.-Y., Park J.-B., Ji S.-H., Kim E.-H., Lee J.-H., Kim H.-S., Kim J.-T. (2025). Comparison of combined intranasal dexmedetomidine and ketamine versus chloral hydrate for pediatric procedural sedation: A randomized controlled trial. Korean J. Anesthesiol..

[31] Oluş F., Babun H. (2025). Sedation applications in pedodontics procedures: Which one should we choose? A retrospective analysis. BMC Oral Health.

[32] Shanmugaavel A.K., Asokan S., Baby J.J., Priya G., Gnana Devi J. (2016). Comparison of Behavior and Dental Anxiety During Intranasal and Sublingual Midazolam Sedation—A Randomized Controlled Trial. J. Clin. Pediatr. Dent..

[33] Wang H., Huang J., Yang S., Zhang X.-f., Yang X., Cui C., Zou C., Li L.-e., Zhang M., Mao M.-f. (2022). Bioavailability and Safety of a New Highly Concentrated Midazolam Nasal Spray Compared to Buccal and Intravenous Midazolam Treatment in Chinese Healthy Volunteers. Neurol. Ther..

[34] Patel M., Vaishnav K., Patel C., Mehta M., Kumar S. (2025). Atomized Intranasal Sedation Efficacy in Children Undergoing Dental Treatment: A Comparative Randomized Clinical Study. Adv. Hum. Biol..

[35] Barends C., den Daas I., Driesens M., Visser A., Absalom A., Colin P. (2023). Development of a pharmacokinetic and pharmacodynamic model for intranasal administration of midazolam in older adults: A single-site two-period crossover study. Br. J. Anaesth..

[36] Behera N.K., Nayak A.K., Lal P., Kumar N., Attar J.R., Dubey A., Dixit H. (2025). Evaluation of Efficiency of Different Sedation Procedures in Pediatric Dental Procedures: A Clinical Study. J. Pharm. Bioallied Sci..

[37] Bahetwar S.K., Pandey R.K., Saksena A.K., Chandra G. (2011). A comparative evaluation of intranasal midazolam, ketamine and their combination for sedation of young uncooperative pediatric dental patients: A triple blind randomized crossover trial. J. Clin. Pediatr. Dent..

[38] Cenzato N., Pasquali L., Menozzi G., Maspero C. (2025). Exploring Conscious Sedation in Pediatric Oral Surgery: A Non-Randomized Clinical Trial on Safety and Efficacy. Children.

[39] Poonai N., Creene C., Dobrowlanski A., Geda R., Hartling L., Ali S., Bhatt M., Trottier E.D., Sabhaney V., O’Hearn K. (2023). Inhaled nitrous oxide for painful procedures in children and youth: A systematic review and meta-analysis. Can. J. Emerg. Med..

[40] Preethy N.A., Somasundaram S. (2021). Sedative and behavioral effects of intranasal midazolam in comparison with other administrative routes in children undergoing dental treatment–a systematic review. Contemp. Clin. Dent..

[41] Johnson E., Briskie D., Majewski R., Edwards S., Reynolds P. (2010). The physiologic and behavioral effects of oral and intranasal midazolam in pediatric dental patients. Pediatr. Dent..

[42] Khole M., Chavhan P., Sajjanar A., Shah S., Salvi P. (2025). Comparative evaluation of efficacy and safety of nitrous oxide and midazolam for conscious sedation in pediatric dental patients: A systematic review and meta-analysis. J. Dent. Anesth. Pain. Med..

[43] Malhotra P.U., Thakur S., Singhal P., Chauhan D., Jayam C., Sood R., Malhotra Y. (2016). Comparative evaluation of dexmedetomidine and midazolam-ketamine combination as sedative agents in pediatric dentistry: A double-blinded randomized controlled trial. Contemp. Clin. Dent..

[44] Liu Y., Li B., Wu X., Xia B., Yang X., Cheng T. (2025). Deep sedation using intranasal dexmedetomidine followed by intravenous propofol for pediatric dental treatment. BMC Oral Health.

[45] Hossenally H., Doughty J. (2022). Intranasal (IN) Midazolam Sedation to Facilitate Dental Treatment for Adults with Autism and Learning Disabilities: An Audit of Efficacy and Safety in Primary Care. SVOA Dent..

[46] Schulz-Weidner N., Hofmann M., Uebereck C., Krämer N., Schlenz M.A., Becker V., Edinger F., Leicht D., Müller M.F., Zajonz T.S. (2025). Interdisciplinary management of patients with special healthcare needs undergoing dental treatment in a tertiary care hospital setting in Germany: A retrospective study. Eur. Arch. Paediatr. Dent..

