# Supplementary material for: Intranasal and Oral Pharmacological Sedation in Pediatric Dentistry: A Thematic Scoping Review of Efficacy, Recovery, and Safety Outcomes
Source: Children (Basel). 2026 Aug 27;13(9):1153. doi: 10.3390/children13091153 (PMC13605337; doi:10.3390/children13091153)
Supplement: Supplementary file 1 [file children-13-01153-s001.zip › Supp_S2_Search_Strategy.pdf]

Supplementary Table S2: Search Strategy

A representative PubMed search string developed including the terms Search: *(((conscious sedation) AND (pediatric dentistry)) AND (oral sedation)) AND (intranasal sedation) Filters: from 2010/1/1 - 2025/11/30 Sort by: Most Recent*

(("conscious sedation"[MeSH Terms] OR ("conscious"[All Fields] AND "sedation"[All Fields]) OR "conscious sedation"[All Fields]) AND ("paediatric dentistry"[All Fields] OR "pediatric dentistry"[MeSH Terms] OR ("pediatric"[All Fields] AND "dentistry"[All Fields]) OR "pediatric dentistry"[All Fields]) AND (("mouth"[MeSH Terms] OR "mouth"[All Fields] OR "oral"[All Fields]) AND ("sedate"[All Fields] OR "sedated"[All Fields] OR "sedating"[All Fields] OR "sedation"[All Fields] OR "sedations"[All Fields])) AND (("intranasal"[All Fields] OR "intranasally"[All Fields]) AND ("sedate"[All Fields] OR "sedated"[All Fields] OR "sedating"[All Fields] OR "sedation"[All Fields] OR "sedations"[All Fields]))) AND (2010/1/1:2025/7/31[pdat])

Translations

conscious sedation: "conscious sedation"[MeSH Terms] OR ("conscious"[All Fields] AND "sedation"[All Fields]) OR "conscious sedation"[All Fields]

pediatric dentistry: "paediatric dentistry"[All Fields] OR "pediatric dentistry"[MeSH Terms] OR ("pediatric"[All Fields] AND "dentistry"[All Fields]) OR "pediatric dentistry"[All Fields]

oral: "mouth"[MeSH Terms] OR "mouth"[All Fields] OR "oral"[All Fields]

sedation: "sedate"[All Fields] OR "sedated"[All Fields] OR "sedating"[All Fields] OR "sedation"[All Fields] OR "sedations"[All Fields]

intranasal: "intranasal"[All Fields] OR "intranasally"[All Fields]

sedation: "sedate"[All Fields] OR "sedated"[All Fields] OR "sedating"[All Fields] OR "sedation"[All Fields] OR "sedations"[All Fields] Search results were imported into Rayyan for screening, and duplicates were removed.

Embase (via Elsevier, Embase.com)

Set	Search terms
**#1**	'child'/exp OR 'adolescent'/exp OR 'pediatrics'/exp OR 'minor (person)'/exp OR (child* OR infant* OR toddler* OR preschool* OR 'pre school' OR kid OR kids OR pediatric* OR paediatric* OR adolescen* OR teen* OR youth* OR juvenile* OR schoolchild* OR minors OR 'young people' OR boys OR girls):ti,ab,kw
**#2**	'sedation'/exp OR 'conscious sedation'/exp OR 'deep sedation'/exp OR 'sedative agent'/exp OR 'anxiolytic agent'/exp OR 'premedication'/exp OR (sedation* OR sedative* OR sedated OR sedating OR 'minimal sedation' OR 'moderate sedation' OR 'procedural sedation' OR 'dental sedation' OR anxiolysis OR anxiolytic* OR premedicat* OR 'pre medication' OR 'behaviour management' OR 'behavior management' OR 'behaviour guidance' OR 'behavior guidance' OR 'pharmacological management'):ti,ab,kw
**#3**	'midazolam'/exp OR 'dexmedetomidine'/exp OR 'ketamine'/exp OR 'chloral hydrate'/exp OR 'hydroxyzine'/exp OR 'promethazine'/exp OR 'diazepam'/exp OR 'triclofos'/exp OR 'melatonin'/exp OR 'clonidine'/exp OR 'pethidine'/exp OR 'sufentanil'/exp OR 'fentanyl'/exp OR 'zolpidem'/exp OR 'buspirone'/exp OR 'propofol'/exp OR 'benzodiazepine derivative'/exp OR (midazolam OR dexmedetomidine OR ketamine OR 'chloral hydrate' OR hydroxyzine OR promethazine OR diazepam OR triclofos OR melatonin OR clonidine OR meperidine OR pethidine OR sufentanil OR fentanyl OR zolpidem OR buspirone OR propofol OR benzodiazepin*):ti,ab,kw
**#4**	'dentistry'/exp OR 'pedodontics'/exp OR 'dental procedure'/exp OR 'dental care'/exp OR 'dental anxiety'/exp OR 'tooth extraction'/exp OR 'oral surgery'/exp OR (dental OR dentist* OR dentistry OR odontolog* OR tooth OR teeth OR 'oral surgery' OR exodontia OR pulpotom* OR pulpectom* OR caries OR 'dental treatment' OR 'oral rehabilitation'):ti,ab,kw
**#5**	'intranasal drug administration'/exp OR 'oral drug administration'/exp OR 'sublingual drug administration'/exp OR 'buccal drug administration'/exp OR (intranasal* OR 'intra nasal' OR transnasal* OR nasal* OR 'per os' OR peroral* OR 'by mouth' OR sublingual* OR buccal* OR transmucosal* OR 'oral administration' OR 'orally administered' OR 'oral route' OR 'oral sedation' OR 'oral premedication' OR atomiz* OR atomis*):ti,ab,kw
**#6**	#2 OR #3
**#7**	#1 AND #6 AND #4
**#8**	#7 AND ([1-1-2010]/sd NOT [1-12-2025]/sd)
**#9**	#8 AND #5

Scopus (Elsevier)

Set	Search terms
**#1**	TITLE-ABS-KEY (child* OR infant* OR toddler* OR preschool* OR "pre school" OR kid OR kids OR pediatric* OR paediatric* OR adolescen* OR teen* OR youth* OR juvenile* OR schoolchild* OR minors OR "young people")
**#2**	TITLE-ABS-KEY (sedation* OR sedative* OR sedated OR "minimal sedation" OR "moderate sedation" OR "procedural sedation" OR "conscious sedation" OR "deep

Set	Search terms
	sedation" OR "dental sedation" OR anxiolysis OR anxiolytic* OR premedicat* OR "behaviour management" OR "behavior management" OR "behaviour guidance" OR "behavior guidance")
#3	TITLE-ABS-KEY (midazolam OR dexmedetomidine OR ketamine OR "chloral hydrate" OR hydroxyzine OR promethazine OR diazepam OR triclofos OR melatonin OR clonidine OR meperidine OR pethidine OR sufentanil OR fentanyl OR zolpidem OR buspirone OR propofol OR benzodiazepin*)
#4	TITLE-ABS-KEY (dental OR dentist* OR dentistry OR odontolog* OR tooth OR teeth OR "oral surgery" OR exodontia OR pulpotom* OR pulpectom* OR caries OR "oral rehabilitation")
#5	TITLE-ABS-KEY (intranasal* OR "intra nasal" OR transnasal* OR nasal* OR "per os" OR peroral* OR "by mouth" OR sublingual* OR buccal* OR transmucosal* OR "oral administration" OR "orally administered" OR "oral route" OR "oral sedation" OR atomiz* OR atomis*)
#6	#1 AND (#2 OR #3) AND #4 AND PUBYEAR > 2009 AND PUBYEAR < 2026
#7	Route-restricted sensitivity set: #6 AND #5

Web of Science Core Collection

Set	Search terms
#1	TS=(child* OR infant* OR toddler* OR preschool* OR pediatric* OR paediatric* OR adolescen* OR teen* OR youth* OR juvenile* OR schoolchild* OR minors)
#2	TS=(sedation* OR sedative* OR "conscious sedation" OR "moderate sedation" OR "procedural sedation" OR "deep sedation" OR "dental sedation" OR anxiolysis OR anxiolytic* OR premedicat* OR "behaviour management" OR "behavior management" OR "behaviour guidance" OR "behavior guidance")
#3	TS=(midazolam OR dexmedetomidine OR ketamine OR "chloral hydrate" OR hydroxyzine OR promethazine OR diazepam OR triclofos OR melatonin OR clonidine OR meperidine OR pethidine OR sufentanil OR fentanyl OR zolpidem OR buspirone OR propofol OR benzodiazepin*)
#4	TS=(dental OR dentist* OR dentistry OR odontolog* OR tooth OR teeth OR "oral surgery" OR exodontia OR pulpotom* OR pulpectom* OR caries)
#5	TS=(intranasal* OR transnasal* OR nasal* OR "per os" OR peroral* OR sublingual* OR buccal* OR transmucosal* OR "oral administration" OR "orally administered" OR "oral sedation" OR atomiz* OR atomis*)
#6	#1 AND (#2 OR #3) AND #4 [Timespan 2010-01-01 to 2025-11-30]
#7	Route-restricted sensitivity set: #6 AND #5

Cochrane Central Register of Controlled Trials

Set	Search terms / syntax as entered
**#1**	MeSH descriptor: [Child] explode all trees OR MeSH descriptor: [Infant] explode all trees OR MeSH descriptor: [Adolescent] explode all trees OR MeSH descriptor: [Pediatrics] explode all trees
**#2**	(child* OR infant* OR toddler* OR preschool* OR paediatric* OR pediatric* OR adolescen* OR teen* OR youth* OR juvenile* OR schoolchild*):ti,ab,kw
**#3**	#1 OR #2
**#4**	MeSH descriptor: [Conscious Sedation] explode all trees OR MeSH descriptor: [Deep Sedation] explode all trees OR MeSH descriptor: [Hypnotics and Sedatives] explode all trees OR MeSH descriptor: [Anti-Anxiety Agents] explode all trees OR MeSH descriptor: [Preanesthetic Medication] explode all trees
**#5**	(sedation* OR sedative* OR "conscious sedation" OR "moderate sedation" OR "procedural sedation" OR "dental sedation" OR anxiolysis OR anxiolytic* OR premedicat* OR "behaviour management" OR "behavior management"):ti,ab,kw
**#6**	(midazolam OR dexmedetomidine OR ketamine OR "chloral hydrate" OR hydroxyzine OR promethazine OR diazepam OR triclofos OR melatonin OR clonidine OR meperidine OR pethidine OR sufentanil OR fentanyl OR zolpidem OR buspirone OR propofol):ti,ab,kw
**#7**	#4 OR #5 OR #6
**#8**	MeSH descriptor: [Dentistry] explode all trees OR MeSH descriptor: [Dental Care for Children] explode all trees OR MeSH descriptor: [Dental Anxiety] explode all trees OR (dental OR dentist* OR dentistry OR odontolog* OR tooth OR teeth OR "oral surgery" OR exodontia OR pulpotom* OR pulpectom* OR caries):ti,ab,kw
**#9**	#3 AND #7 AND #8 with Publication Year from 2010 to 2025, in Trials
