# Supplementary material for: Intranasal and Oral Pharmacological Sedation in Pediatric Dentistry: A Thematic Scoping Review of Efficacy, Recovery, and Safety Outcomes
Source: Children (Basel). 2026 Aug 27;13(9):1153. doi: 10.3390/children13091153 (PMC13605337; doi:10.3390/children13091153)
Supplement: Supplementary file 1 [file children-13-01153-s001.zip › Supp_S3_Excluded_Studies with reason.pdf]

Supplementary Table S3: Excluded Studies with Reasons

NO.	Authors/ Year	Study Design	Excluded studies with reasons
1	Mangione, Bdeoui et al. 2020	A retrospective study	Excluded because of age group above 18
2	Castaño Novoa, Limeres Posse et al. 2024	Pilot study	Excluded because no outcome on the pharmacological approach
3	Maeda, Kita et al. 2005	cross-sectional study	Excluded because no outcome on the pharmacological approach, unspecified age group and year of publication
4	Orellana, Martínez-Sanchis et al. 2014	cross-sectional study	Excluded because no outcome on the pharmacological approach and year of publication
5	Konia 2016	Case report	Excluded because no outcome on the pharmacological approach, type of the study (case report), and age group
6	Messieha, Cruz-Gonzalez et al. 2008	Retrospective study	Excluded because no outcome on the pharmacological approach, year of publication and age group.
7	Loo, Graham et al. 2008	Randomized trial	Excluded because of age group above 18 and year of publication
8	Ouchi and Sugiyama 2015	Retrospective study	Excluded because no outcome on the pharmacological approach and age group
9	Asahi, Omichi et al. 2013	Case report	Excluded because no outcome on the pharmacological approach and year of publication

10	Marshall, Sheller et al. 2008	Cross-sectional study	Excluded because of years of publication and reported outcomes were not supportive of IN sedation
11	Hulland and Sigal 2000	Cross-sectional	Excluded because it was a non-pharmacological approach and year of publication
12	Ohtawa, Yoshida et al. 2019	Cross-sectional study	Excluded because the unspecified age group, no primary outcome on pharmacological approach
13	Park and Sigal 2008	Case report	Excluded because of the type of the study (case report), no primary outcome on pharmacological approach and year of publication
14	Santos, Valle et al. 2015	Descriptive quantitative study	Excluded because of unspecified age group and no primary outcome on pharmacological approach
15	Loo, Graham et al. 2009	Descriptive quantitative study	Excluded because of unspecified age group and year of publications
16	Mac Giolla Phadraig, Griffiths et al. 2018	Cross-sectional survey	Excluded because of age group and they have not mentioned IN sedation
17	Chang, Patton et al. 2014	Prospective study	Excluded because not related to primary outcome
18	Prabhu, Nunn et al. 2008	Qualitative study	Excluded because of unspecified age group
19	Ohtawa, Tsujino et al. 2012	Retrospective survey	Excluded because of unspecified age group
20	Maeda, Tomoyasu et al. 2012	Retrospective cohort study	Excluded because of unspecified age group, no primary outcome on pharmacological approach
21	Douglass and Glassman 2013	Narrative review	Excluded because of the type of the study (review article)

22	Tilja, Rajavaara et al. 2019	Retrospective study	Excluded because of no mention about the pharmacological interventions.
23	Asahi, Kubota et al. 2009	Prospective study	Excluded because of unspecified age group, no primary outcome on pharmacological approach
24	Wang, Lin et al. 2012	Review article	Excluded because of the type of the study (review article)
25	Stoner, J. B. et al. 2006	Retrospective study	Excluded because of unspecified age group, no primary outcome on pharmacological approach
26	Zink A.G. et al. 2016	Pilot study of RCT	Excluded because of unspecified age group, no primary outcome on pharmacological approach
27	Komsic, J. et al. 2020	Development of educational model	Excluded because of age group
28	Ferriera, F. et al 2020	Case report	Excluded because of the type of the study (case report)
29	Hossenally, H. and Daughty, J. 2021	Retrospective study	Excluded because of unspecified age group, no primary outcome on pharmacological approach
30	Lim, GXD and Boyle, CA. 2020	Review literature	Excluded because of the type of the study (Review literature)
31	Marinho MA. et al. 2022	Retrospective	Excluded because of unspecified age group, no primary outcome on pharmacological approach
32	Jo et al. 2017	Case report	Excluded because of the type of the study (case report)
33	Penna et al. 2023	RCT, double blinded	Excluded because of unspecified age group, no primary outcome on pharmacological approach
34	Machado et al. 2020	Narrative review	Excluded because of the type of the study (review)

35	Faulks et al. 2007	Cross-sectional descriptive	Excluded because of unspecified age group, no primary outcome on pharmacological approach, and year of publication
36	Almutawwif et al. 2025	Review article	Excluded because it was a review article
37	Berry 2025	Review	Excluded because it was a review article
